# Notes and News

**Published:** 1886-11-27

**Authors:** 


### Notes and News.

Collected and Communicated.

On Thursday, the 18th inst., Lady Brabazon and the members of the Kyrle Society gave a musical entertainment to the patients at the National Hospital for diseases of the heart and paralysis, Soho Square.

Sir Richard Webster has decided to apply the £10,000 bequeathed to him by the late Mr. Lucas Walker "for the promotion of scientific and literary research," to the foundation of a studentship (of the annual value of £200 to £300) and prizes, for the encouragement of original research in pathology. These will be open to candidates of either sex by competition in Cambridge or London.

The employés of Sir W. G. Armstrong and Co., Elswick Works, have contributed a sum of £224 5*s.* 1*d.* to the Hospital Saturday Fund. This is in addition to their ordinary collection on behalf of the local medical charities, which this year amounted to £155 7*s.* 7*d.*

By the death of Lady Wilson the Royal College of Surgeons becomes entitled to a legacy of about £230,000, left by her husband, the late Sir Erasmus Wilson.

The Manchester charities have benefited largely under the will of the late Alderman Thomas Rose, whose funeral took place the other day. In all, he has left £22,000 to local benevolent institutions, including £10,000 to the Royal Infirmary, £5,000 to the Salford Hospital, £2,000 to St. Mary's Hospital for Women, and £1,000 each to the Eye Hospital, Lock Hospital, and Clinical Hospital.

The committee of "The People's Contribution Fund" earnestly invite subscriptions of one shilling and upwards to assist in the establishment and endowment of a bed (to be called "The Queen's Bed") at the North London or University College Hospital, in honour of the jubilee year of Her Majesty's reign. Any donations will be gratefully received by the honorary secretary, Mr. Newton H. Nixon, at the Hospital, Gower Street, W.C.

At a recent meeting of the General Court of Guy's, the vacancy in the list of physicians to this hospital, caused by the lamented death of Dr. Moxon, was filled up by the appointment of Dr. Goodhart, of Weymouth Street, Portland Place. Dr. Goodhart has been for some years an assistant physician at Guy's. He also holds the post of physician to the Evelina Hospital for Children. Dr. Goodhart is one of the ablest and most courteous of physicians, and we tender him our hearty congratulations on his promotion.

The Children's Hospital at Paddington Green affords a notable instance of rapid growth. Founded only a year or two since, the hospital already numbers nearly thirty beds, and the poor of Paddington and the district use its out-patient department in constantly increasing numbers. There are few examples of a Children's Hospital which have met with more encouraging success.

A gentleman has promised to give five hundred pounds towards the enlargement of the Bristol Eye Hospital (a much needed work), if the balance required can be collected by February next. The necessary expenditure is computed at £3,500, and contributions to "The Bristol Eye Hospital Extension Fund" are earnestly requested. They may be sent to the treasurer, Mr. C. D. Cave, Stoneleigh House, or to the Old Bank, Bristol.

Lord Meath, President of the Dublin Hospital Sunday Council, while "asking the forbearance of those who have already proved themselves to be such liberal supporters of this benevolent work," ventures to recall to their memories the fact "that the consensus of public opinion in all civilised nations of Europe points to the indispensable necessity of maintaining hospitals, either by private voluntary subscriptions or by subsidies from the public funds of their countries." "Should any, however," he continues, "if such a one exists, argue this matter on a selfish and lower ground, it must clearly convince him that all classes,—upper, middle, and lower,—would inevitably be sufferers in their own persons and families through the spread of infectious diseases, and from the inefficiency of a medical profession deprived of its proper schools of instruction." So clear and concise a reminder of the individual as well as general benefit derived from hospital work is well worthy of notice, and should bring home to all classes the necessity of maintaining our public charities.

The Netherfield Road Hospital, Liverpool, is now erected, and was inspected by the officials of the Corporation on the 14th inst. As our readers will remember, it is a Doecker temporary hospital, and has been supplied by the Doecker Hospitals and Hut Factory, 35, Eastcheap. The material used is paper-felt, lined with jute and fixed on wooden frames, which are easily put together and do not require any nails or screws. Several Doecker huts were sent out to Egypt for the use of our troops during the Soudan campaign, their great advantage being that they resist extremes of temperature.

The new erection at Netherfield Road, which is intended as a fever hospital, contains two wards, each capable of accommodating six patients, and there is also a special room for the nurses. It is heated by ventilating stoves, and will be lighted by an improved gas system.

The Laura Franklin Free Hospital for Children, in New York, is nearly completed, and was to be ready for the reception of patients about the middle of the present month. It has been built by Mr. and Mrs. Franklin

Delane in memory of their niece, and the total cost, including endowment, will be about 300,000 dollars. The kitchen, laundry, and boiler room are all detached from the main building. It is intended for children of from two to twelve years old, and will accommodate fifty patients.

---

The Kilmarnock Infirmary has been presented by Mrs. Finnie, of Springhill House, with a new ambulance waggon for the conveyance of patients suffering from accident or illness not of an infectious character. It is fitted to carry on one side two or three people on stretchers or cots, while several persons can be accommodated on a broad folding seat, which extends the length of the vehicle on the opposite side. Rugs and a full equipment of medical and other stores are supplied in the waggon. This is a most useful gift, and one in every way deserving the gratitude of the inhabitants of Kilmarnock.

---

The new wing of the Bootle Borough Hospital is rapidly approaching completion, and will be ready for the reception of patients in the course of a month or two. Funds for furnishing this new wing, and defraying the cost of the necessary alterations in the original building, are urgently required.

---

The following vacancies are announced this week, the amount of the salary being placed in each case after the name of the institution :—

**Honorary Medical Officers.**

Glamorganshire Infirmary, Cardiff. Ophthalmic Surgeon.
Bradford Infirmary. Physician of five years' standing.

**Resident Medical Officers.**

Hull Royal Infirmary. Junior Assistant House Surgeon. Fully qualified.—£50, with board.
Ancoats Hospital, Manchester. Junior Visiting Surgeon.—£60, with board.
Glasgow Hospital for Sick Children. House Surgeon. Registered.—£60, with board.
Whitechapel Infirmary. Superintendent, doubly qualified.—£250 and apartments.
Kensington Dispensary. Doubly qualified.—£125, and apartments.
Wallasey Dispensary, Cheshire. Doubly qualified House Surgeon.—£120, and furnished residence.

**Trained Nurses.**

City of London Chest Hospital, Victoria Park. Several.—£20, with uniform.
Glamorganshire Infirmary, Cardiff. Head.—£28, with uniform.
Kidderminster Infirmary. Medical and Surgical, Night.—£20, with uniform.
Brompton Consumption Hospital. One.
Worcester Nurses' Institution. Several.—£25, with uniform.
Kent Nursing Institution, West Malling. Several.—£24 to £30, with uniform.

---

The late Colonel Brocklehurst, who has been a munificent supporter of the Macclesfield Infirmary, has, we are informed, left the sum of £1,000 to the Endowment Fund, free from legacy duty. The late Mr. Edwin Chadwick, another friend of the Macclesfield Infirmary, has also made a handsome bequest. The Governors propose, with these welcome additions to the Endowment Fund, to further extend the resources of their deserving charity, so that it may be better able to cope with the increasing demands made upon it.

---

The annual report, just issued, shows how useful a Society the St. John Ambulance Association is. Among the numerous services rendered by pupils of the Association, in all parts of the kingdom, may be noted with interest the treatment of over 600 cases at the ambulance station in "Old London." An instructive synopsis of the work carried on by the Metropolitan Ambulance Corps is also given in the report.

---

The steady increase in the number of pauper lunatics during the past ten years is matter for regret. In 1875, the number of lunatics chargeable to the poor-rates was 54,571, and there has been a regular yearly increase, so that in 1885 there were no less than 69,700, or 27.7 per cent. more than in 1875. Moreover, these figures do not include 1,670 pauper lunatics chargeable to counties or to boroughs, so that the real total of insane poor in all England is 71,370.

---

Some idea of the density of the population of East London may be gathered from the statement, that at the Stratford Dispensary during last year 9,524 patients were admitted for treatment, 38,594 persons received medicine, and 5,207 patients were visited at their own homes, in addition to 8,251 cases of emergency which received immediate relief. The Stratford Dispensary is very popular, under the management of Dr. Francis the resident house-surgeon, and the present idea is to place the new hospital at the back of the Dispensary, in West Ham Lane, thus following the example of Greenwich, where the Miller Memorial Hospital has been built on the circular system, at the back of the Royal Kent Dispensary in the Greenwich Road.

---

A meeting was held in Camden Hall on the 18th inst., Lieut.-Colonel Milman presiding, when a committee was formed to raise funds in the district towards the cost of the new buildings of the Great Northern Central Hospital, now being erected in the Holloway Road. Several medical practitioners of the neighbourhood testified to the great necessity of the work, that part of North London being practically unprovided with hospital accommodation. The following donations have been received during the current month (November) :

| | |
|---|---|
| *For the General Fund.* | |
| Amalgamated Friendly, Temperance, and Trade Societies .. .. .. .. .. .. .. | £80 |
| Ladies' Association of the G. N. C. Hospital .. .. | 50 |
| Miss Chippendale .. .. .. .. .. .. | 5 |
| *For the Building Fund.* | |
| North London Amalgamated Temperance Societies .. | 18 |
| Crosby Lockwood, Esq. .. .. .. .. .. .. | 30 |
| John L. Pound, Esq. .. .. .. .. .. .. | 20 |
| Total .. .. .. .. | £203 |

---

The medical classes in connection with Aberdeen University were opened on Wednesday, the 22nd ultimo. The number of students has been this session largely augmented by a more than ordinary number of beginners. In fact, the number of candidates at the preliminary examination was this year greater than ever it has been. The most notable introductory lectures were those of Professor MacWilliam, the newly-installed Professor of Physiology, and of Professor Hamilton. Dr. MacWilliam, who was introduced to his future students by Principal Geddes, gave a very clear and interesting account of the rise and growth of Physiology; while Dr. Hamilton gave a graphic description of a visit he paid during last recess to the laboratories of Pasteur and Koch. It has appeared as one of a series of articles on the burning question of the prophylactic treatment of hydrophobia in the *Scotsman*. In every respect the prospects of the Aberdeen medical school are bright.

---

Not only the school, however, but medical science in the North of Scotland has sustained a distinct loss in the untimely death of Dr. Davidson, Professor of Materia Medica in the University. A man of marked ability and strong mental grip, Dr. Davidson was beloved alike by his colleagues, his students, and his patients. As a Professor the description of him which has been universally recognised as the truest by those who knew him in that capacity is, that to the last he was a student. Big-minded, large-hearted, ever ready to do a kindly deed, his memory will ever be lovingly cherished by those whose good fortune it was to know him.

---

In addition to being Professor of Materia Medica, Dr. Davidson held the post of Ophthalmist to the infirmary and dispensary. For both these offices candidates are beginning to present themselves, and it is rumoured that in neither case will the appointment be long delayed. Dr. Matthew Hay, Professor of Medical Jurisprudence, is conducting the Materia Medica class *pro tempore.*

---

We are indebted to Mr. Henry F. Aveling, the Clerk to the Paddington Guardians, for an account of the new Infirmary adjoining the workhouse in the Harrow Road. It has been built from the plans of Messrs. A. and C. Harston of Leadenhall Street, and contains accommodation for 284 patients, with an average cubic space per head of from 886 feet in the large wards, to 1,170 in the small ones, and a superficial area per bed of 75 square feet. It has cost about £40,000, including furniture and fittings, and the Guardians are to be commended for preparing a scheme for nursing the sick in this Infirmary on a fairly liberal basis. The matron is Miss Jane E. Styring, and there is one nurse to every 13.6 beds. It is satisfactory to note that the medical superintendent, Dr. Savill, has expressed himself satisfied with the arrangements and buildings.

---

Few societies deserve better of the public than the Kyrle Society for bringing beauty home to the people. Founded in 1877, with the motto, "To the utmost of our power," its members have done so much for the fulfilment of their objects as to more than justify the motto. Recently they held an exhibition in the Cromwell Road, which was full of interest and instruction. It consisted of works of art prepared by the members for distribution amongst hospitals, workhouses, and other institutions. Some of the exhibits give proof of real artistic merit, and showed the artists to possess skill of a high order. The Kyrle Society undertakes the distribution of flowers and plants, not only to institutions, but to the poor in their own homes. The supply is, however, neither full nor regular, and suitable plants for indoor decoration have never yet been offered. How many of those who shudderingly flee from London during the time of fog and gloom, to bask in the lovely sunshine of the south of France, could find a pleasing novelty of occupation in selecting, once a week, a hamper of flowers, and despatching it by parcels post to Miss E. S. Busk, Kyrle Society, 14, Nottingham Place, London, W. We know we have readers resident in the Riviera, and we hope they may be moved to try the luxury of the new departure here suggested to them.

---

There seems just now to be a rage amongst provincial hospital authorities for applying any windfalls, such as legacies, private donations, etc., which may come in their way, to pulling down the existent buildings, and erecting new ones in their place. We cannot help thinking that they would do well to take the advice recently given by Mr. S. Aldred to the governing body of the Yarmouth Hospital, and invest unexpected legacies for the permanent benefit of their institutions. It is, of course, sometimes necessary to increase the accommodation, but surely this object might be equally well and more economically effected by adding to, rather than completely rebuilding, the premises already in existence.

---

The authorities of the Blackheath and Charlton Cottage Hospital are of opinion that the loyalty of their neighbours would find its most suitable expression in contributions towards the erecting of a new and larger building in place of the present one, to be called the Jubilee Hospital. The Earl of St. Germans has offered a site at a nominal rental of £5, and another gentleman has promised £200 towards the cost, which, for a building capable of accommodating thirty patients, is estimated at £2,000. The existing premises have been open three years, and consist of two dwelling-houses converted into one. We are sorry to see this proposal made. The Miller Memorial Hospital, the general hospital for south-east London, ought rather to be extended and made adequate to the wants of this large district. It would be a fatal error to unduly multiply small hospitals in suburban London.

---

The inquest on Mrs. Peek, who died, according to the medical evidence, in the infirmary of St. Pancras Workhouse from acute rheumatism, accelerated by the fracture of her arm, throws a side light on the nursing of that institution which reveals a condition of affairs that it was believed and hoped did not prevail in the present day within the walls of metropolitan poor-law infirmaries at any rate. Mrs. Peek wishing to be lifted out of bed, two very aged women, one of whom is described as "the help," and the other as an aged and infirm patient, came to her assistance, although she was a very heavy woman. As a natural consequence their strength proved insufficient, and she fell, fracturing her arm and dislocating her shoulder. We agree with the jury in the rider which they appended to their verdict, in which they call upon the guardians to provide younger and able-bodied persons to attend to the patients in the St. Pancras Workhouse Infirmary. We have every confidence that this rider will bear good fruit, and we shall therefore refrain from further criticisms, which would certainly not be out of place under the circumstances.

